# Opening the dialogue: A preliminary exploration of hair color, hair cleanliness, light, and motion effects on fNIRS signal quality

**DOI:** 10.1371/journal.pone.0304356

**Published:** 2024-05-23

**Authors:** Mitchell Holmes, Daniel Aalto, Jacqueline Cummine

**Affiliations:** 1 Faculty of Rehabilitation Medicine, University of Alberta, Edmonton, Alberta, Canada; 2 Faculty of Medicine and Dentistry, Neuroscience and Mental Health Institute, University of Alberta, Edmonton, Alberta, Canada; 3 Faculty of Rehabilitation Medicine, Department of Communication Sciences and Disorders, University of Alberta, Edmonton, Alberta, Canada; 4 Institute for Reconstructive Science in Medicine (iRSM), Misericordia Community Hospital, Edmonton, Alberta, Canada; Air University, PAKISTAN

## Abstract

**Introduction:**

Functional near-infrared spectroscopy (fNIRS) is a promising tool for studying brain activity, offering advantages such as portability and affordability. However, challenges in data collection persist due to factors like participant physiology, environmental light, and gross-motor movements, with limited literature on their impact on fNIRS signal quality. This study addresses four potentially influential factors–hair color, hair cleanliness, environmental light, and gross-motor movements–on fNIRS signal quality. Our aim is to raise awareness and offer insights for future fNIRS research.

**Methods:**

Six participants (4 Females, 2 Males) took part in four different experiments investigating the effects of hair color, hair cleanliness, environmental light, and gross-motor movements on fNIRS signal quality. Participants in Experiment 1, categorized by hair color, completed a finger-tapping task in a between-subjects block design. Signal quality was compared between each hair color. Participants in Experiments 2 and 3 completed a finger-tapping task in a within-subjects block design, with signal quality being compared across hair cleanliness (i.e., five consecutive days without washing the hair) and environmental light (i.e., sunlight, artificial light, no light, etc.), respectively. Experiment 4 assessed three gross-motor movements (i.e., walking, turning and nodding the head) in a within-subjects block design. Motor movements were then compared to resting blocks. Signal quality was evaluated using Scalp Coupling Index (SCI) measurements.

**Results:**

Lighter hair produced better signals than dark hair, while the impact of environmental light remains uncertain. Hair cleanliness showed no significant effects, but gross motor movements notably reduced signal quality.

**Conclusion:**

Our results suggest that hair color, environmental light, and gross-motor movements affect fNIRS signal quality while hair cleanliness does not. Nevertheless, future studies with larger sample sizes are warranted to fully understand these effects. To advance future research, comprehensive documentation of participant demographics and lab conditions, along with signal quality analyses, is essential.

## 1 Introduction

Neuroimaging techniques play a pivotal role in elucidating the intricacies of brain function and organization, offering invaluable insights into the neural underpinnings of cognition, behavior, and neurological disorders. As the field of neuroscience continues to evolve, the demand for advanced neuroimaging tools capable of providing high-resolution spatial and temporal information has become increasingly pronounced. In this context, the ability to acquire high-quality neuroimaging data efficiently is essential for maximizing research productivity and advancing scientific knowledge. Streamlining the neuroimaging process not only accelerates research progress but also enhances the reproducibility and reliability of findings, thereby strengthening the foundation of neuroscience research.

**Fig 1:**
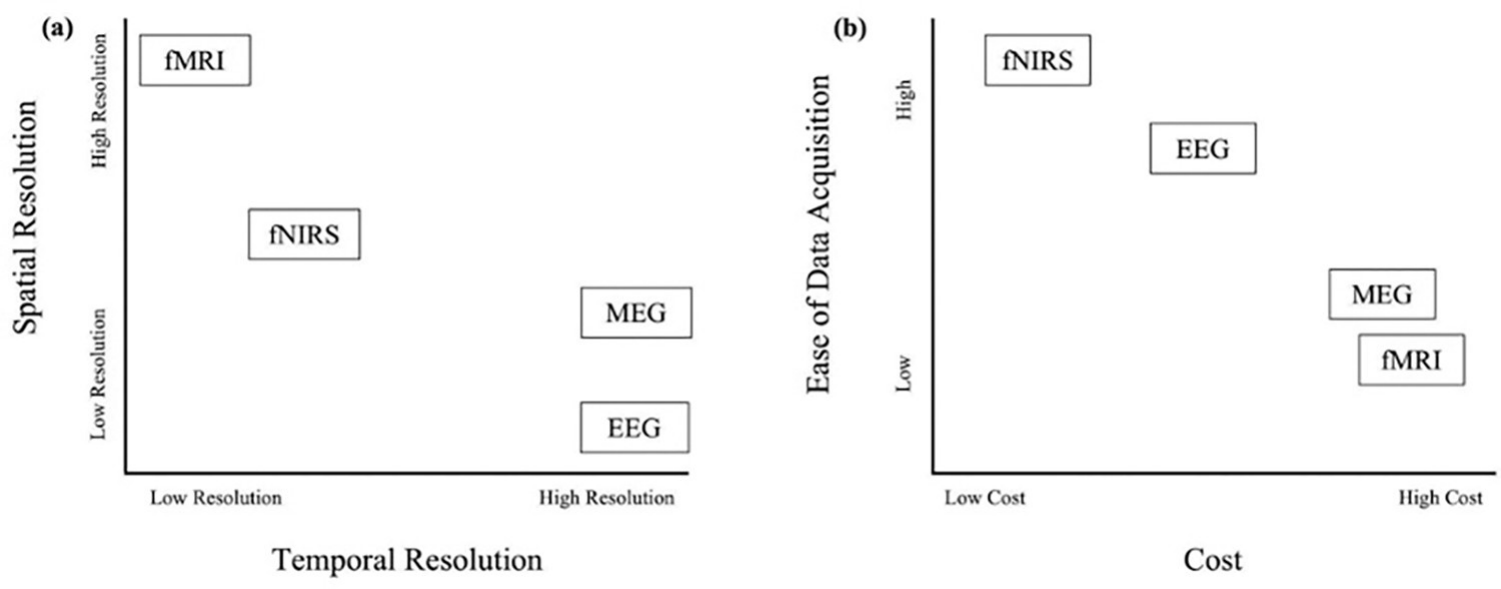
Temporal vs. spatial resolutions and cost vs. ease of data acquisition among imaging methodologies. (a) A schematic representing the relative strengths and weaknesses of temporal and spatial resolution across imaging modalities. (b) A schematic representing the relative differences in costs and ease of data acquisition presented across imaging modalities.

Among available neuroimaging techniques, functional near-infrared spectroscopy (fNIRS) distinguishes itself with practical advantages that enhance imaging efficiency. With superior temporal resolution than functional magnetic resonance imaging (fMRI) [1] and more robust spatial resolution relative to magnetoencephalography (MEG) or electroencephalography (EEG) [2,3], fNIRS offers researchers a balanced approach to pinpointing the timing and location of brain activation during cognitive tasks. Moreover, fMRI, MEG, and EEG are all inherently susceptible to motion-generated artifacts [4] and require specially designed environments for data acquisition, such as shielded rooms [5–7]. These drawbacks, coupled with the non-portability of the techniques, participant contraindications, costs, and the need for specialized technicians [1], have prompted an increase in the use of fNIRS methodology. fNIRS provides a safe, cost-effective, and logistically feasible alternative for studying brain-related phenomena [1,8]. For a visual representation of how fNIRS compares to fMRI, MEG, and EEG in terms of resolution, ease of data acquisition, and costs, refer to Fig 1.

However, unlike fMRI, MEG, and EEG, fNIRS has not yet been subjected to the same level of scrutiny and documentation of its limitations. Several review papers have begun to fill this knowledge gap: Orihuela-Espina et al. [9] provide a comprehensive outline of the various factors that could influence fNIRS signals; Hocke et al. [10] sought to provide researchers with instructions on how to implement the appropriate processing pipelines and what to expect from them; Cieśla et al. [5] and Pinti et al. [2], worked to inform future fNIRS researchers on the suitability of the technology in its application to different research applications. However, while these sources have been invaluable in their synthesis of many factors that impede high-quality fNIRS signals as well as providing substantial guidance and recommendations to optimize signal quality, an updated account of the challenges associated with fNIRS is warranted. We begin by briefly summarizing the documented factors that are suggested to play a role in hindering the quality a researcher can achieve from an fNIRS-based paradigm, namely participant population and physiology factors, environmental factors, and motion factors [9].

Physiological factors of a population, such as age [11–13], sex [11,14], skin color [15], properties of the hair [16], medical history [9], and the use of medications are capable of compromising an fNIRS signal [17,18]. Relevant to the current study is the impact of hair color on fNIRS signals. The characteristics of an individual’s hair, including color and density, have the potential to impact the signal. For instance, darker hair colors can reduce signal intensity by 20–50% while hair density may affect the stability of the optodes’ positioning [16]. Notably, there is no documentation about the impact of hair and scalp cleanliness on fNIRS signals, although such reports have been noted in the EEG literature [19,20]. While good experimental work will report on, and/or control for, each of the factors mentioned above, Experiment 1 (E1) and 2 (E2) of the current paper provides an account of the impact of hair color and hair cleanliness on fNIRS signals to better understand the repercussions of these underrepresented factors in fNIRS experiments.

Environmental conditions such as room humidity, temperature, and ventilation are said to influence the fNIRS signal quality, as electronics tend to work at their best when these conditions are controlled to stay at normal levels (e.g., room temperature) [9]. Fortunately, these factors are relatively easy to mitigate in laboratory-based settings. What is a bit more challenging to mitigate is the ambient light, whether natural or artificial, which is proposed to affect the quality of the fNIRS signal, particularly when there is an inefficient connection between the optodes and the skin of the participant [9]. In the absence of a tight optode-skin coupling, the photodetectors can detect the surrounding light and contaminate the signal. Collecting data in a dark room [12,21,22] or covering the cap with an opaque material [21,23,24] is said to reduce the effects that environmental light can have on the signal. We explore the impacts of environmental light and current recommendations in Experiment 3 (E3).

Being a portable system, fNIRS allows researchers to collect data in natural environments. Thus, it is often advertised as a method that can be used for studies that involve unrestrained, gross motor movements (see Pinti et al. [25] for a review of fNIRS being used in naturalistic settings). However, sudden or large body movements carry the potential to create artifacts in neuroimaging data, especially when such movements disrupt the placement of the fNIRS optodes [26]. Ensuring the optodes are securely attached to the fNIRS cap can limit their potential to shift, but it is important to recognize that if the optodes are held too tightly against the participant’s head, it can cause discomfort and even pain, leading to confounding variables or premature termination of data collection. Although not as significant, even smaller movements such as jaw motions from overt speech and reading tasks have the potential to affect the resulting signal quality [27]. Motion correction algorithms, such as Wavelet Filtering and Spline Interpolation which filter out noise, high-frequency spikes, and baseline shifts [10], can be used as a means to account for the motion-generated artifacts, but to an extent that has yet to be fully realized. Experiment 4 (E4) investigates the effects that large body movements have on the resulting fNIRS signal.

Therefore, despite the attractions of the advantages fNIRS has to offer, we have encountered several factors that have hindered the process of both data collection and analysis, and for which there is little documented literature about the magnitude of these roadblocks on signal quality. Thus, our aim in this work was to conduct a preliminary investigation of four factors–hair color, hair cleanliness, environmental light, and motion–which could in principle have an effect on fNIRS signal quality. Our research is intended to raise awareness within the fNIRS community about these understudied factors and initiate a discourse on which ones warrant more robust exploration to uncover the full extent of their potentially adverse effects.

## 2 Research questions, hypotheses & design

This research will address four questions:

Q1. Does hair color affect the quality of fNIRS signals?
Q2. Does hair cleanliness affect the quality of an fNIRS signal?
Q3. Does environmental light, both artificial and natural, affect the quality of an fNIRS signal?
Q4. Does participant motion, such as walking, turning the head, or nodding the head, affect an fNIRS signal?

Our hypotheses are as follows:

Darker hair colors (e.g., black) will produce a lower quality due to undersaturation (i.e., receivers not detecting enough light), while people with no hair, will produce a lower quality signal due to oversaturation (i.e., receivers detecting too much light).
Freshly cleaned (i.e., shampooed) hair/scalp will produce a higher quality signal when compared to 1, 2, 3, and 4 days after cleaning.
Artificial light will have less of an effect on signal quality than natural light, while darker conditions will produce a better signal.
Increased movement with the fNIRS cap on will negatively affect the signal quality (i.e., the signal will get worse as the movement continues).

We opted to employ a single case experimental design (SCED) to address the aforementioned research questions. A SCED provides several advantages that were important for the current study [28,29]: 1) *Controlled Manipulation*: SCEDs allow researchers to systematically manipulate the independent variable (i.e., movement) while controlling extraneous variables (i.e., light), providing a high level of internal validity. This control enhances our ability to draw causal inferences about the effects of the manipulation; 2) *Individual Analysis*: SCEDs focus on the behavior of individual participants, which is advantageous when studying a methodology where variability across individuals is high. This approach can provide valuable insights into individual differences in responding to manipulations; 3) *Experimental Rigor*: SCEDs involve multiple phases of baseline and manipulation conditions, along with replication within the same participant. This design rigor enhances the reliability and validity of the findings; 4) *Sensitive to Change*: SCEDs are often more sensitive to detecting changes in the dependent measure over time compared to group designs. This sensitivity is particularly useful when evaluating manipulations that may produce relatively small changes in the dependent measure; 5) *Flexibility*: SCEDs offer flexibility in terms of design parameters, allowing researchers to tailor the design to the specific needs of the study and the characteristics of the participant. This flexibility can enhance the ecological validity of the research; 6) *Ease of Implementation*: SCEDs are often relatively simple to implement, requiring fewer participants and resources compared to group designs. This can make SCEDs more feasible, particularly in settings with limited resources or when studying hard-to-reach populations; 7) *Ethical Considerations*: SCEDs involve fewer participants, which reduced ethical concerns related to participant burden given the systematic nature of Experiments 2, 3 and 4, in particular (see also [30–32] for similar designs using fNIRS).

## 3 Methods

### 3.1 Participants

Six participants were recruited using purposive and convenience sampling methods. Five (Females = 4; Males = 1) were selected on the basis of hair color (i.e., black hair, brown hair, red hair, blonde hair, no hair) and one (Male = 1) was selected based on his availability to come to the lab for five consecutive days without washing his hair in between each day. Given that four experiments were conducted in this study, not all participants completed each one. Refer to Table 1 to for details on which experiments each participants completed. All participants were 18+, identified as cis-gender, and had white-colored skin. Data collection began on February 17^th^, 2023 and was completed by April 5^th^ 2023. An honorarium in the form of a $10 gift card was offered to all participants for their time spent participating. This research study has been approved and conducted in accordance with the Research Ethics Board (REB) at the University of Alberta (Pro00128407).

**Table 1:** Experiments completed by each participant.

Participant	Experiments					
	E1 [Color]	E2 [Cleanliness]	E3 [Light]	E4 [Walk]	E4 [Headturn]	E4 [Headnod]
P001	✓					
P002	✓		✓	✓		
P003	✓		✓	✓	✓	✓
P004	✓		✓	✓	✓	✓
P005		✓				
P006	✓		✓	✓	✓	✓

This table depicts the experiments that each participant undertook. With the exception of participant P005, each individual was chosen based on their hair color. They were then allowed to complete what other experiments they could based on their availability and comfort level.

### 3.2 Materials

**Fig 2:**
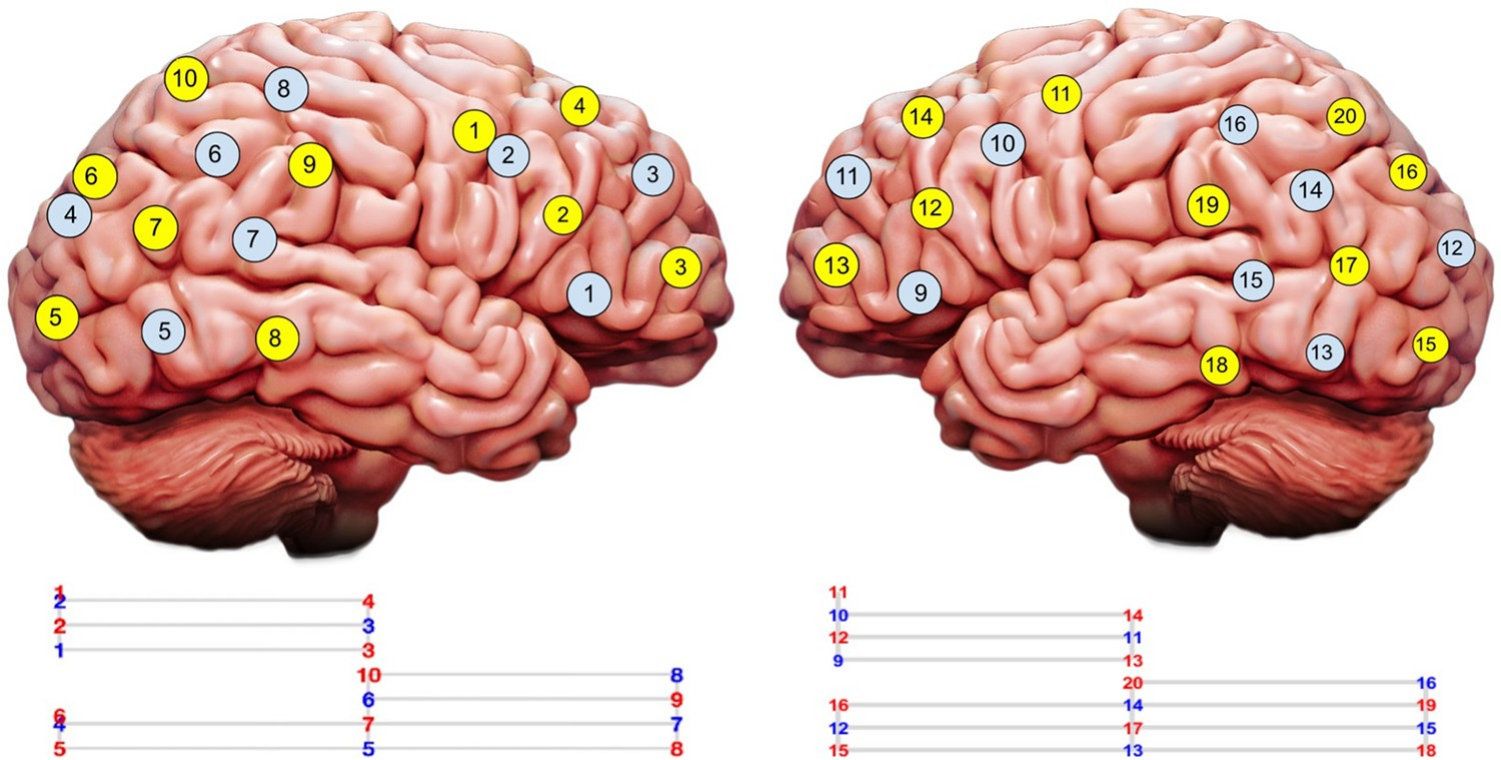
The Optode template visualized on the brain. Yellow and blue circles indicate transmitter and receiver positions, respectively.

The fNIRS device used in this study was the 1907 version of the Artinis Brite24 in conjunction with the program Oxysoft, the software used to record and handle the data measured with fNIRS [33]. As a non-invasive imaging technology, fNIRS assesses activity in the brain through the use of near-infrared light corresponding to an approximate wavelength range of 650 nm to 900 nm [2]. Within this range, hemoglobin absorbs infrared light emitted by the transmitters and is then detected by receivers–the resulting change in light absorption detected by the receivers provides us with a method of measuring the hemodynamic changes in the blood [34]. Since functional brain activity is understood to be associated with changes in oxyhemoglobin and deoxyhemoglobin, fNIRS devices are specifically measuring the concentrations of these two molecules [2]. The Brite24 is a two-device system, with each device containing identical optodes to be placed on either side of an individual’s head, enabling the concurrent acquisition of oxygenated hemoglobin concentrations in the brain. Each device has 10 transmitter optodes and 8 receiver optodes, resulting in a total of 45 optode pairs (i.e., channels). The transmitters on each device operated at wavelengths of 760 nm and 850 nm. These channels were distributed across the right and left hemispheres following the template seen in Fig 2. The right-side array contained two short channels, one posterior and one anterior, which are used to remove extracortical hemodynamic activity from superficial layers of tissue, such as the scalp, during post-processing [35–37]. Due to the constraint of having only two available short channels, their placement was informed by literature indicating higher correlation of extracortical data between symmetrical brain regions across hemispheres than within ipsilateral regions [36]. Consequently, this arrangement allows posterior extracortical data from the right side to be removed from right and left posterior regions in the post-processing stages, while the anterior extracortical data from the right side is removed from right and left anterior regions. fNIRS devices were connected via Bluetooth to a computer where the signals were recorded. In addition to the fNIRS recording equipment, the stopwatch app on an iPhone XR was used to time the experimental and control blocks (Table 2).

**Table 2:** Experimental and control blocks performed in each experiment.

	Experimental Block	Control Block
E1 & E2 Color/Cleanliness	Finger-tap	Rest
E3 Environmental Light	Finger-tap	Rest
E4 Walking	Walk	Rest/Finger-tap
E4 Headturn/Headnod	Turn/Nod	Rest/Finger-tap

Participants alternated between experimental block and control blocks for the instructed amount of time followed by the control block and repeated the sequence as many times as instructed.

### 3.3 Procedure

After reading and signing a consent form, participants were measured for the fNIRS cap and its placement while seated in front of a computer. Placement of the cap was guided by the International 10–20 positioning system, beginning with two measurements of the head (i.e., tragus to tragus, inion to nasion) to find the mid-point of the skull (Cz). The transmitters and receiver optodes were then placed into their designated optode holders. Once the optodes were in place, data acquisition (DAQ) values, which indicate the percentage of light that is being received by the receivers, were assessed to determine the initial signal quality of each channel. If the DAQ values are close to 100%, then the signal is oversaturated, if the values are close to 0% the signal is undersaturated. In both cases, the corresponding channel will be colored red, indicating that the signal for that channel is of poor quality [33]. If the signal is within the acceptable range, the channel is colored white (the default range for what is considered a good signal varies among manufacturers of fNIRS software and equipment and can be found in their respective materials. In our study, we utilized OxySoft, the proprietary NIRS recording software developed by Artinis Medical Systems. However, we were unable to locate specific details regarding the range of signal quality that the program uses within the software. This presents another challenge to fNIRS data acquisition). In the case that the channel was undersaturated, the associated optodes would be lowered closer to the participants’ skin to increase the signal. If the channel remained red, the optodes would be removed from their holders so hair could be moved out of the way to increase the contact between the optode and the scalp before returning the optode. If the signal is oversaturated, the opposite was done: the optodes would be moved further away from the scalp within their holders or, if need be, the optode would be removed to adjust hair and decrease the contact between the optode and the scalp. To maintain consistency in setting up the fNIRS cap, the same researcher administered the setup procedure for every participant. Procedures specific to each experimental condition are described in more detail under each experiment below.

A maximum of 30 minutes was allotted to obtain the best signal quality possible, with the timer starting once the cap had been placed on the participant’s head. This decision was influenced by several practical considerations, such as the battery life of the fNIRS system and the amount of time we had with each participant. Moreover, we are highly sensitive to our participants’ comfort and well-being during data collection. In past research, participants have reported discomfort and even pain when wearing an fNIRS cap for extended periods. This discomfort can affect their performance on cognitive tasks and, consequently, the quality of the brain signals recorded. In theory, it is possible that we could have obtained a better signal had we continued to ‘tweak’ the optodes past the 30-minute mark, however, the benefits are not outweighed by the additional time as some participants may choose to end data collection early. After the signal for all channels was obtained or the time limit was reached, the number of channels that were within the range of what the software indicated were good quality (i.e., the channels that were colored white) and how long it took to achieve that level of quality was noted.

The experimental procedures were then begun. To test the independent variables (i.e., hair color, hair cleanliness, environmental light) in E1, E2, and E3, participants completed a finger-tapping task (i.e., repeatedly touching each finger to the thumb one by one). Finger tapping was selected for its extensive documentation and frequent utilization in both fMRI [38] and fNIRS research [4,30–32,35,39–41], presenting a straightforward motor task that is easily executed and standardized across participants. In E4, participants completed a series of gross motor movements (i.e., walking, turning the head, nodding the head) to test their effects on signal quality (see Lacerenza et al. [32] for a similar design used to monitor the hemodynamic response function during forward and backward walking). All experiments used a block-design, similar to those found in the fNIRS literature [30–32,35,39,40]. Details specific to each experimental procedure can be found below.

### 3.4 SCI analysis

Signal quality analysis was performed with the Quality Testing of Near Infrared Scans (QT-NIRS) toolbox. QT-NIRS toolbox was developed by Hernandez and Pollonini [42] (https://github.com/lpollonini/qt-nirs), which calculates the Scalp Coupling Index and peak power for every five-second time frame. QT-NIRS conducts a post-experiment assessment of the signal quality based on the SCI, which is determined by calculating the correlation between the cardiac signal present in oxy- and deoxygenated hemoglobin. For each task and condition, the QT-NIRS output consists of a bar graph of the channels that exceeded the threshold (i.e. channels that had good data quality). The SCI and peak power threshold for E1, E2, and E3 were set to 0.5 and 0.1, respectively. In E4, the SCI was conducted at a threshold of 0.5 and 0.8 while the peak power threshold was set to 0.1. The number of good-quality channels were counted and reported for each participant in each task.

## 4 Experiment 1 (E1)–hair color

### 4.1 Methods

#### 4.1.1 Participants

Participants (N = 5; blonde, red, brown, black, no hair; Female = 4; Male = 1) were 18+, identified as cis-gender, and had white-colored skin. They were recruited via a purposive sampling method to select participants that matched the hair color required (Table 1).

#### 4.1.2 Procedure

A between-subjects block design was used to test and compare the differences in fNIRS signal quality between black hair, brown hair, red hair, blonde hair, and no-hair conditions. Participants were instructed to alternate between 10 s of finger-tapping with the right hand (experimental block) and 10 s of rest (control block) for two minutes, resulting in a total of six experimental and six control blocks. Differences in signal quality were compared between the five different hair-colored individuals.

### 4.2 Results

It was found that for each participant, the full 30 minutes of set-up were needed to obtain the best signal. Channel quality was assessed via the collection software (as noted above) and QT-NIRS SCI to determine consistency. The SCI analysis determined that P001, who had brown hair, had 32 channels above the 0.5 threshold. The number of good quality channels determined by the collection software for this participant were not able to be obtained. P002, who had blonde hair, had 38 channels within the optimal range according to the SCI. Conversely, the collection software indicated that there were only 36 good quality channels. The SCI showed us that our participant with no hair, participant P003, had 31 channels of optimal quality while the collection software indicated 26. For P004, our red-haired participant, 28 good quality channels were obtained as reported by the SCI. The collection software portrayed 26 good quality channels. Finally, SCI analysis showed that participant P005 ended up with 33 good quality channels and the collection software showed that they had 22 good quality channels. P005 was our black-haired participant. Please refer to Table 3 for a summary of these results.

**Table 3:** Number of good quality and bad quality channels in E1.

Participant	Good Quality Channels	Bad Quality Channels
P001 (brown hair)	32	13
P002 (blonde hair)	38(36)	7(9)
P003 (no hair)	31(26)	14(19)
P004 (red hair)	28(36)	17(9)
P006 (black hair)	33(22)	12(23)

Numbers outside of the brackets are the number of channels determined by SCI analysis. Those in brackets are determined by the collection software.

To examine the generalizability of these findings to a larger sample, an exploratory analysis was subsequently conducted on a dataset from an unrelated study in our lab (the research study was approved and conducted in accordance with the Research Ethics Board (REB) at the University of Alberta (Pro00096774)). Participants included 28 adults (Females = 21; Age = 24.2 SD = 5.9) who completed a simple word naming task while seated in a chair. Individuals were categorized with respect to hair color (i.e., black, brown, blonde, red) and hair thickness (i.e., thick vs. average). The SCI associated with a frontal optode pair was categorized as ‘good’ or ‘poor’ if it passed the threshold of 0.5. A Chi-square analysis was conducted and we found that participants with black hair were more likely to have poor SCIs compared to brown, blonde and red, X^2^ (3) = 10.3, *p* = 0.016. Individuals with thick hair were more likely to have poor SCIs compared to individuals with average/fine hair, X^2^(1) = 4.1, *p* = 0.043.

## 5 Experiment 2 (E2)–hair cleanliness

### 5.1 Methods

#### 5.1.1 Participants

The participant (N = 1) was an 18+, cisgender male, with brown colored hair and white skin. He was recruited via convenience sampling at the University of Alberta (Table 1).

#### 5.1.2 Procedures

A within-subjects block design was used to test and compare the differences in fNIRS signal quality after consecutive days of not washing the hair. The participant was instructed to alternate between 10 s of finger-tapping with the right hand (experimental block) and 10 s of rest (control block) for two minutes, resulting in a total of six experimental and six control blocks. The task was then repeated with the left hand. This procedure was repeated every day for five successive days, where the participant washed their hair with shampoo on the first day of measurements and did not rinse or wash their hair with water or shampoo again until after the last measurement on the fifth day. Differences in signal quality were compared between the five subsequent days. In addition, the hemodynamic response function (HRF) at the dorsolateral prefrontal cortex (DLPFC) was plotted for each day and compared.

### 5.2 Results

It was found that for each day of data collection, the full 30 minutes were needed to obtain the best signal. The number of channels that the collection software indicated were within an acceptable range for each of the five days were 26, 41, 42, 42, and 45, respectively. Conversely, SCI analysis revealed 38, 42, 41, 41, and 42 channels with good quality for each respective day. Visual inspection of the SCI bar graphs also indicated that the majority of good quality channels were at 100%. Days 1 through 5 had 33, 42, 40, 36, and 42 channels at 100%, respectively. Please refer to Table 4 for a summary of these results.

**Table 4:** Number of good quality and bad quality channels for participant P005 in E2.

	Good Quality Channels	Bad Quality Channels
Day 1	38(26)	7(19)
Day 2	42(41)	3(4)
Day 3	41(42)	4(3)
Day 4	41(42)	4(3)
Day 5	42(45)	3(0)

Numbers outside of brackets are the number of good quality channels determined by SCI analysis. Those in brackets are determined by the collection software.

## 6 Experiment 3 (E3)–environmental light

### 6.1 Methods

#### 6.1.1 Participants

Participants (N = 4; Female = 3; Male = 1) were 18+, identified as cis-gender and had white-colored skin. The participants were selected from those who had previously participated in E1 and agreed to take part in E3 as well (Table 1).

#### 6.1.2 Procedures

E3 consisted of five different conditions: windows uncovered and overhead lights on (i.e., sunlight and artificial lights), windows uncovered and overhead lights off (i.e., sunlight only), windows covered and overhead lights on (i.e., artificial lights only), windows covered and overhead lights off (i.e., no lights), and windows covered and overhead lights off with an opaque covering (i.e., a black toque or a black bag) placed overtop the optodes (i.e., zero light). The order in which the conditions were presented was randomized for each participant. A within-subjects block design was used to test and compare the differences between each light condition for every participant. Participants were instructed to alternate between 10 s of finger-tapping with the right hand (experimental block) and 10 s of rest (control block) for two minutes, resulting in a total of six experimental and six control blocks. One exception was participant P003, who was instructed to alternate between 30 s of finger-tapping with the right hand and 30 s of rest for three minutes, resulting in a total of three experimental and three control blocks. The task was repeated for each light condition and the differences in signal quality were compared between each condition.

#### 6.1.3 Materials

The overhead lights used for the artificial light condition were fluorescent. In the natural light condition, three windows to the participant’s right would be opened to allow daylight to enter the room. A black cotton toque or black bag would be used for the opaque covering condition. Please refer to Table 5 for information on how each light condition was achieved.

**Table 5:** Experimental conditions of E3.

Condition	Windows	Overhead Lights	Opaque Covering
Sunlight^a^ + Artificial	Open	On	No
Sunlight	Open	Off	No
Artificial	Closed	On	No
No Light	Closed	Off	No
Zero Light^b^	Closed	Off	Yes

^a^The brightness level of the sunlight conditions is described to be in accordance with a Sunny-16 rule for calculating light without a light meter (https://shootitwithfilm.com/what-is-the-sunny-16-rule-and-why-should-you-learn-it/#:~:text=The%20Sunny%2016%20Rule%20is,the%20ISO%20of%20your%20film).

^b^A black cotton toque was used to cover the fNIRS optodes for participants P002, and P003. Participants P004 and P006 had the fNIRS optodes covered by a black bag.

### 6.2 Results

As depicted in Table 6, P002 exhibited the highest number of good quality channels in the sunlight condition, whereas the lowest count was observed in the sunlight + artificial condition. P003 demonstrated the most favorable number of good quality channels in the sunlight and artificial, no light, and zero light conditions. For P004, the highest number of good quality channels was observed in the no-light condition, contrasting with the poorest performance in the artificial condition. P006 showcased the highest good quality channel count in the sunlight + artificial and sunlight conditions, while presenting the lowest count in the zero light condition.

**Table 6:** Number of good quality channels across each light condition determined by SCI.

Participant	Light Conditions				
	Sunlight + Artificial	Sunlight	Artificial	No Light	Zero Light
P002	33	38	35	35	36
P003	32	31	26	32	32
P004	21	21	7	22	7
P006	36	36	35	35	31

The table depicts the number of channels that the SCI determined to be above the threshold of 0.5 for each participant across light conditions.

## 7 Experiment 4 (E4)–motion

### 7.1 Methods

#### 7.1.1 Participants

Participants (N = 4; Female = 3; Male = 1) were 18+, identified as cis-gender and had white-colored skin. The participants were selected from those who had previously participated in E1 and agreed to take part in E4 as well (Table 1).

#### 7.1.2 Procedures

Participants performed three movements: head-nodding (i.e., looking up and down repeatedly), head-turning (i.e., turning the head left and right) and walking (i.e., walking back and forth between two points marked on the floor 6 m apart). The order in which the conditions were presented was randomized for each participant. A within-subjects block design was used to test if large, repeated movements have an effect on signal quality. Additionally, the movement tasks were compared to the other experiments that each participant performed to determine whether gross motor movements had a greater effect on signal quality than when the participants were sitting still.

In the head-nodding task, participants P004 and P006 were instructed to alternate between 30 s of nodding (experimental block) and 30 s of rest (control block) for four minutes, resulting in a total of four experimental and four control blocks. This pattern was replicated in the head-turning task. P003 also performed the head-nodding and head-turning tasks but replaced the resting period with a finger-tapping task. P002 chose not to participate in the head-nodding or head-turning tasks due to the discomfort of wearing the fNIRS cap. In the walking task, participants P004 and P006 were instructed to alternate between 30 s of walking (experimental block) and 30 s of rest (control block) for four minutes, resulting in a total of four experimental and four control blocks. P002 and P003 were instructed to alternate between 60 s of walking and 30 s of finger tapping, resulting in four experimental and four control blocks. Differences in signal quality were then compared between each condition.

### 7.2 Results

At a threshold of 0.5, SCI analysis showed participants P002, P003, P004, and P006 initially had 38, 31, 28, and 33 good quality channels before the movement tasks. The collection software reported 36, 26, 36, and 22 channels. By the end of the last movement task, P002, P003, and P004 had 27, 26, and 7 good quality channels, while P006 increased to 34 channels. According to the collection software, only P002 and P004 lost good quality channels (29 and 26), while P003 and P006 gained good quality channels (30 and 23). At a threshold of 0.8, SCI analysis showed P002, P003, P004, and P006 started with 27, 31, 15, and 31 good quality channels and ended with 9, 27, 0, and 14, respectively. See Table 7 for a summary of these results.

**Table 7:** Number of good quality channels before and after the motion tasks.

Participants	Channels Before Motion Tasks			Channels After Motion Tasks			Difference		
	SCI 0.5	SCI 0.8	CS	SCI 0.5	SCI 0.8	CS	SCI 0.5	SCI 0.8	CS
P002	38	27	36	27	9	29	-11	-18	-7
P003	31	31	26	30	27	30	-1	-4	+4
P004	28	15	36	7	0	26	-21	-15	-10
P006	33	31	22	34	14	23	+1	-17	+1

CS = Collection Software; (-) = loss in good quality channels; (+) = gain in good quality channels.

**Fig 3:**
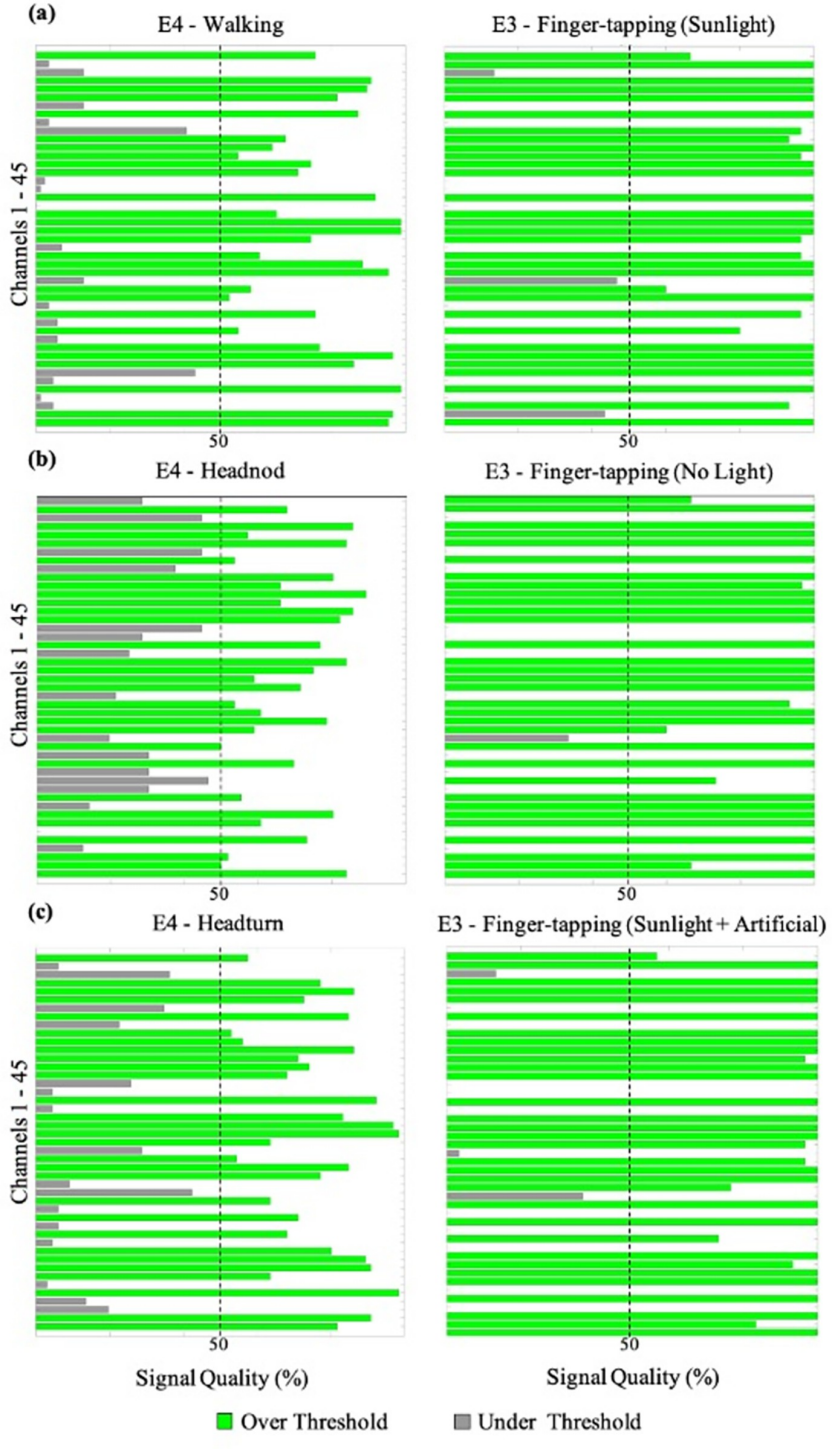
SCI analysis of E4 motion tasks vs. E3 finger-tapping tasks for P003. (a) The walking task compared to a randomly selected task from E3 (finger-tapping). (b) The head-nod task compared to a randomly selected task from E3 (finger-tapping). (c) The head-turn task compared to a randomly selected task from E3 (finger-tapping).

**Fig 4:**
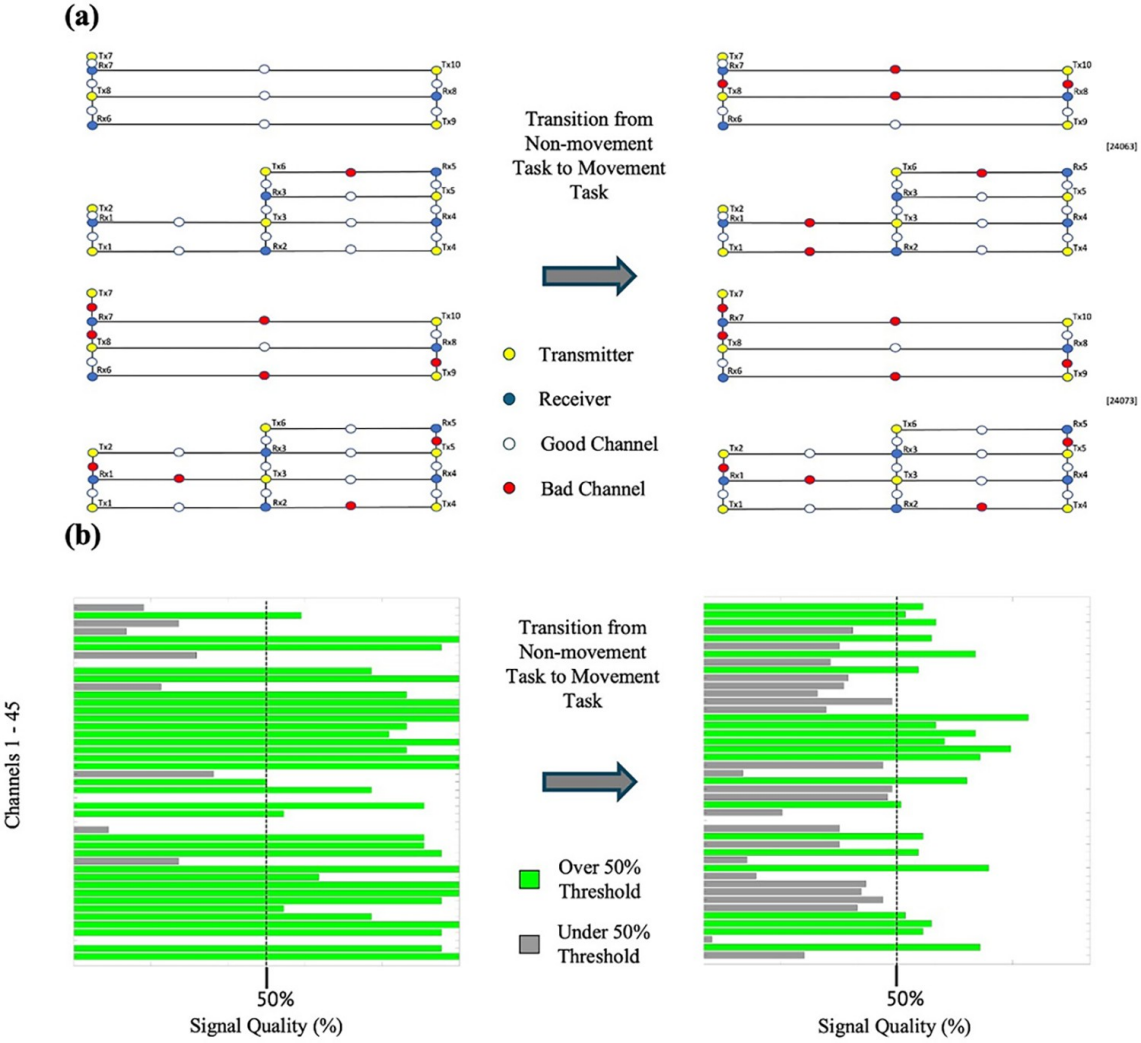
Demonstration of how gross motor movements affect fNIRS signal quality in P002. (a) The transition from a non-gross movement task to a gross movement task by the collection software. (b) before and after the transition by the SCI.

**Fig 5:**
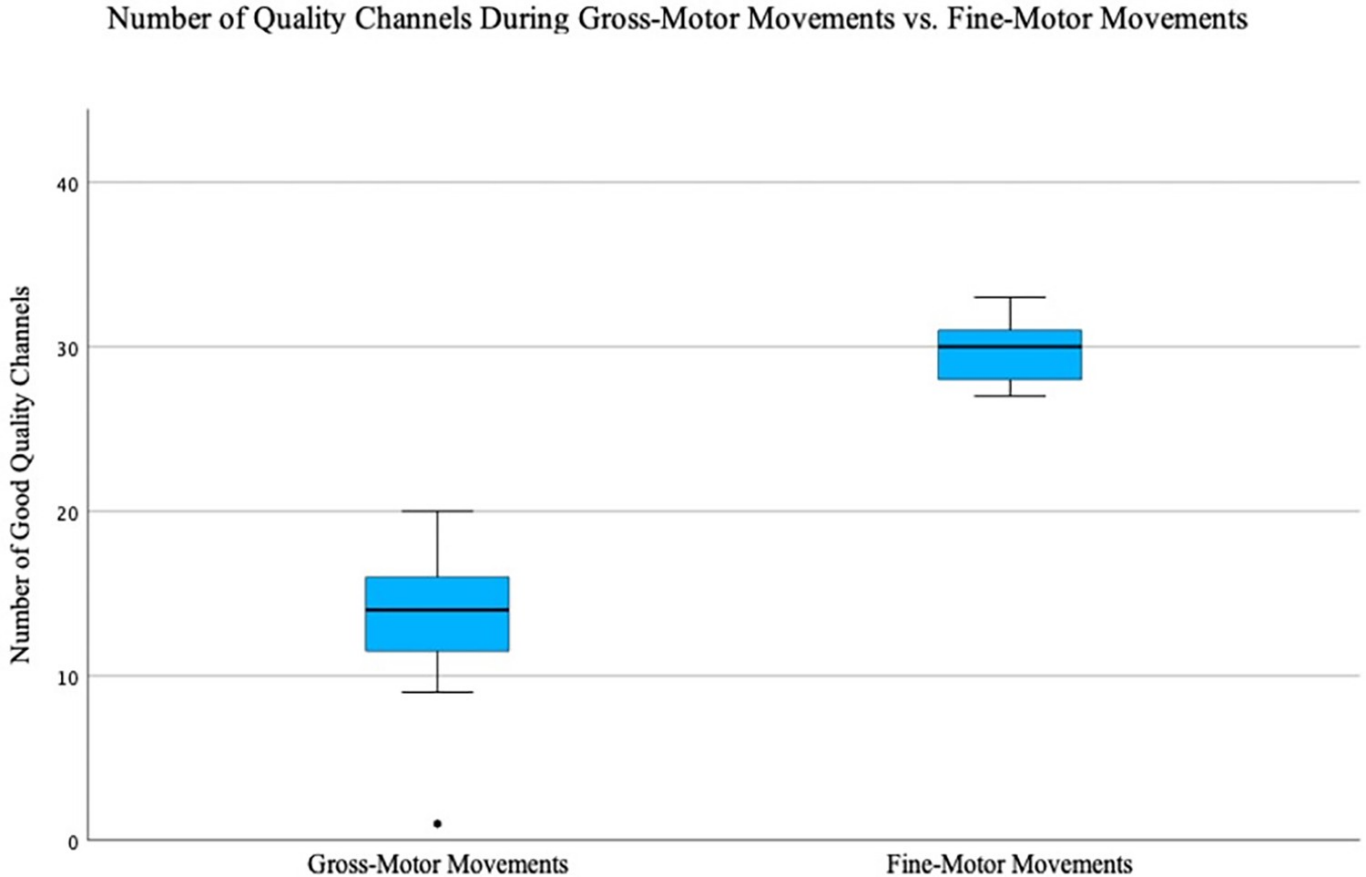
Gross-motor movements vs. fine-motor movements. An independent-samples Kruskal-Wallis nonparametric test was used to evaluate whether the number of good quality channels differed between gross-motor movement tasks (i.e., head-nodding, head-turning, walking) and fine-motor movement tasks (i.e., finger-tapping). The test yielded a significant result of *p* < .001.

In addition to changes in the number of good quality channels for each of the three movement tasks, we also observed how the quality of the fNIRS signal changed between tasks that required participants to display fine motor movements (i.e., finger-tapping) and gross movements (i.e., walking, head-nodding, head-turning). For participants P002 and P006, transitioning from finger-tapping to gross motor tasks led to a signal quality decrease according to the SCI. Conversely, when E4 preceded E3 (as seen in P003), an increase in signal quality was observed. Fig 3 illustrates these changes in P003 by comparing SCIs of walking, head-nodding, and head-turning tasks to three randomly selected conditions from E3. Furthermore, Fig 4 demonstrates a decrease in overall signal quality, as indicated by SCI, during a transition from E3 to E4 for participant P002. A Kruskal-Wallis non-parametric test revealed significant differences (*p* < .001) in the number of good quality channels (SCI threshold = 0.8) between tasks requiring participants to sit and those involving gross-motor movements (Fig 5).

## 8 Discussion

In this work, we explored the impact of hair color, hair cleanliness, natural and artificial light, as well as gross motor movement tasks on the quality of fNIRS signals. During data collection, for each participant, we required the full 30 minutes to obtain the best quality signal possible. This is in line with a remark from Orihuela-Espina et al. [9], who describes the time-consuming nature of obtaining good quality signals through optode adjustment. Even still, only on one occasion (P004—Day 5) did we obtain what our collection software deemed to be a perfect signal (i.e., all channels were of high quality/white). In our data, lighter hair color produced a more robust quality signal compared to darker hair color. However, hair cleanliness and lighting conditions did not have a systematic effect on signal quality. Finally, movement of multiple sorts, markedly influenced signal quality. We discuss the implications of each of these findings in further detail below.

### 8.1 Considerations for initial fNIRS quality screening

Related to the initial optode placement for signal quality was the discrepancy we found between the collection software quality assessment and QT-NIRS SCI analysis. In E1, E2, and E4, when comparing the number of quality channels identified by SCI against those identified by the collection software, we observed only one instance in which the two quality assessments showed agreement: P003 with an SCI threshold of 0.5 after completion of the motor tasks in E4. We suspect that this issue is due to the fact that we are unable to determine the default range of signal quality the collection software uses. Yet these thresholds have crucial implications when setting up and adjusting the optodes on a participant. For example, as seen on Day 5 of E2, the collection software indicated that we had 100% signal strength. But using the offline QT-NIRS program, we observed that this was not the case, as the SCI determined three channels were below the threshold of 0.5 –an arguably liberal threshold, as a threshold of 0.8 is often promoted [42]. Therefore, researchers may opt to carry on with data acquisition without knowing that the channel that corresponds to their targeted area is not actually receiving the quality of signal they believe it is. In order to correct this discrepancy, researchers will have to determine the default settings of their fNIRS equipment and software, which would also require manufacturers to make this information more readily available. Alternatively, there are other programs that could be utilized, such as PHOEBE, which calculates real-time SCI during data collection, giving researchers confidence that the channels of interest are of high quality throughout the experiment [43]. However, these programs come with their own limitations. For example, challenges such as calibrating various programs to work simultaneously with one another can become overly complicated and increase the risk of researcher or software error. Further, while we can be hopeful there is some basic reliability among the classification of high/low quality of fNIRS signals, the extent to which offline programs (i.e., QT-NIRS) and online programs (i.e., PHOEBE) are channel-by-channel consistent in signal quality ratings is not yet established.

### 8.2 Considerations for population and physiological factors

Somewhat in line with our hypothesis for E1, we found that hair color did impact fNIRS signal quality with the blonde-haired condition exhibiting the highest number of good-quality channels through SCI analysis. Anecdotally, these results are consistent with our general findings in the lab (i.e., it is easier to obtain high-quality fNIRS signals with blonde, light brown). Our examination of a larger sample, which categorized participants according to both hair color and thickness, yielded significant findings reinforcing the impact of hair color on signal quality. Specifically, we observed that lighter hair colors and thinner hair were more likely to be associated with better signal quality compared to black and thick hair. Nevertheless, further research is warranted to fully characterize the impact of hair properties on fNIRS signals, including hair length, presence/absence of dyes and other hair products, etc. To add further clarity to this discussion, we also must consider the findings from E2 on hair cleanliness.

The outcomes from E2 did not align with our initial hypothesis, suggesting that refraining from washing hair and allowing scalp oils to accumulate did not weaken the fNIRS signal. However, this participant, who had brown hair, displayed consistently higher signal quality than any of the participants in E1 –had he been included in the hair color experiment, brown hair would be the top ranking. Thus, it is crucial for researchers to record the hair color of their participants when gathering demographic information. This becomes particularly important when a researcher must exclude a portion of data from a dataset when the signal quality does not meet the threshold. In such cases, it is imperative to note whether the excluded subset primarily consists of individuals with a particular hair color. By excluding an entire hair color from our studies, we risk introducing bias and limiting the generalizability of our findings to the broader population. Such consequences have already become evident in existing literature. In one example, researchers resorted to selectively sampling only males with hair lengths shorter than 1 cm, aiming to mitigate hair-related obstacles entirely [39]. Hence, documenting hair color, along with other hair-related exclusionary criteria, will serve as a vital aspect of the continued exploration of this factor.

**Fig 6:**
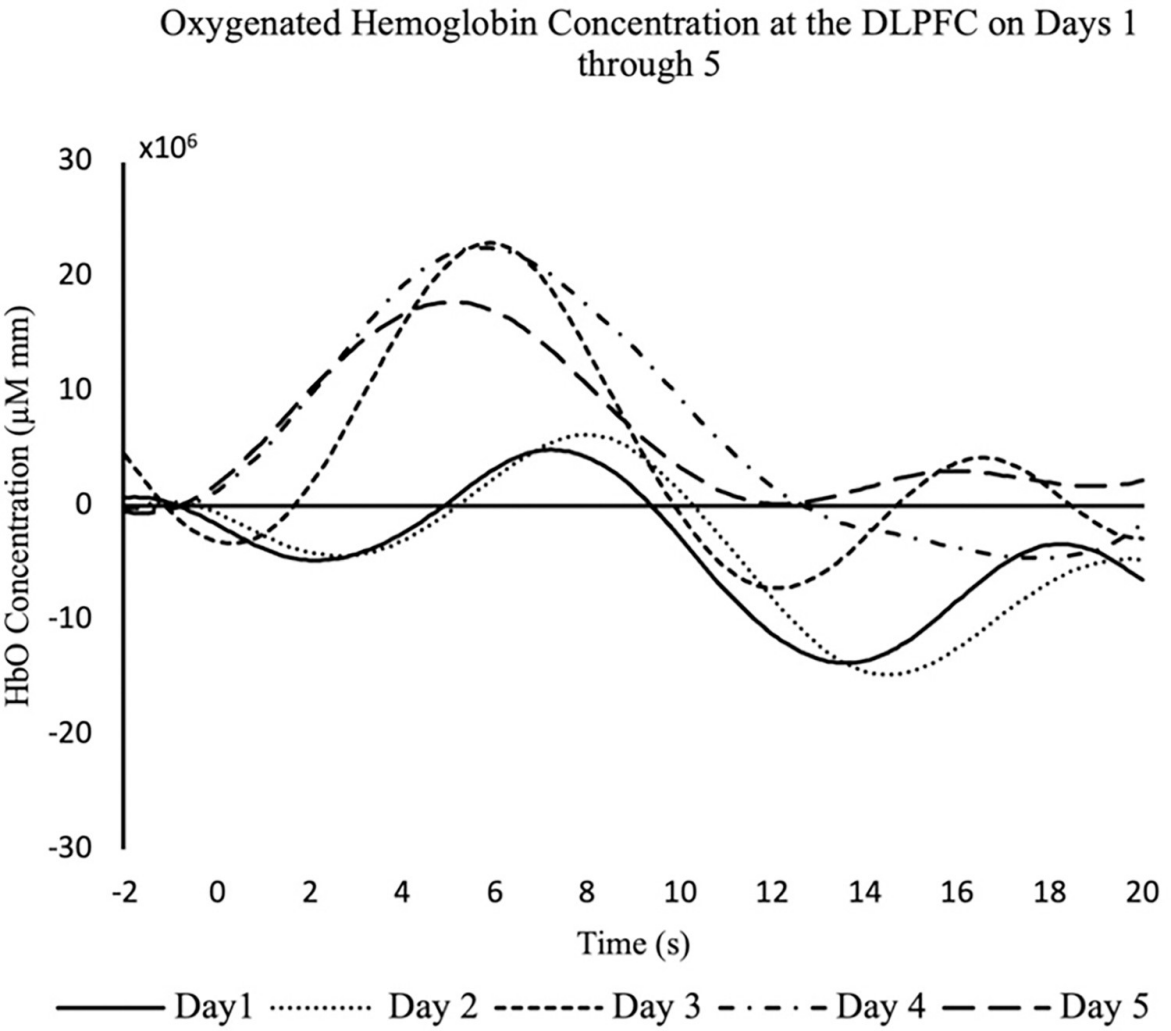
HRF plot of oxygenated hemoglobin at the DLPFC of the hair cleanliness condition. The participant performed a finger-tapping task for 10 s followed by a 10 s rest for 2 minutes every day for five days. HRF curves were then plotted based on the average oxygenated hemoglobin concentration for each block and measured in μM mm. The concentrations were then plotted against time measured in seconds.

Notably, the study designs used in E2 and E3 provide useful information regarding test-retest/reliability of the fNIRS signal. With respect to E2, we were able to consistently measure activity in the DLPFC during finger tapping across 5 days (Fig 6). This finding, while secondary to the initial aims of the experiment, provides useful information about the robustness of the fNIRS signal over time. Indeed, we chose finger tapping as our task of interest as this is a consistent and easy motor task to implement, which has also been recorded extensively in previous literature [4,30–32].

### 8.3 Considerations for environmental factors

In E3 we suspected that either the no light or zero light (i.e., a black toque or black bag placed over the fNIRS cap) conditions would portray a higher number of good quality channels compared to the conditions with more environmental light. However, the results were not consistent with this hypothesis. In fact, there seemed to be little variation between the number of good quality channels within each subject (Table 6). This is with the exception of participant P004, who we are considering to be an outlier because we believe there may have been an external factor that caused such a drastic change between conditions. Across participants, no one condition seemed to affect the signal quality substantially more than another.

Although the findings may not provide conclusive evidence, there is a likelihood that light is influencing the fNIRS signal. This assumption arises from the observation that the number of channels is changing between conditions, despite being careful not to touch or move the cap and being sure to conduct each condition consecutively and immediately after one another. If light had no impact, a consistent number of quality channels would be expected across all conditions. Furthermore, previous literature consistently indicates that light influences fNIRS signals [9,25,39,44]. However, gauging the precise extent of this influence poses a challenge, as it is not common for researchers to document the ambient light conditions during data collection or the resultant light-induced noise in data processing. In cases where ambient light is acknowledged, researchers commonly opt to shield fNIRS sensors with opaque coverings, such as black cloth or aluminum foil, to avoid the impact of environmental light altogether [12,21,24]. Although this is a sensical strategy, we are left without useful data that could aid us in understanding this effect. Therefore, due to the varying channel outcomes in E3, and to the underrepresentation of this issue in the current literature, we suggest that light conditions may indeed play a role in affecting the fNIRS signal quality, warranting further investigation with a larger sample size to ascertain its significance.

### 8.4 Considerations for motion factors

The most robust findings in this study emerged during E4, the movement tasks. A distinct shift in signal quality became evident when comparing fine-motor movements (e.g., finger-tapping) to gross-motor movements (e.g., walking, head-nodding, head-turning). In contrast to tasks involving seated participants tapping their fingers, gross motor movements in E4 exhibited a significant reduction in signal quality, as highlighted in Fig 4. Whether conducted before or after tasks in E3, the signal quality in E4 decreased consistently, suggesting that this decline is indeed attributed to the impact of gross motor movements. This is further supported by the overall decreasing trend in the number of good quality channels after the completion of the movement tasks (Table 7). These findings align with current literature, which indicates that even minor movements can influence fNIRS signal quality. For example, studies have demonstrated that movements of the jaw during overt reading or speech tasks may potentially disrupt signal outcomes [27] thereby underscoring fNIRS sensitivity to motion-induced signal degradation.

Moreover, we observed a substantial difference between the number of high-quality channels at SCI thresholds of 0.5 versus 0.8. While we initially adopted a fairly liberal threshold of 0.5 for conservative findings, a threshold 0.8 has been promoted for fNIRS data analysis [42]. Given the compelling argument presented by E4 suggesting a decrease in signal quality at a threshold of 0.5, we extended our exploration to scrutinize the findings under an SCI of 0.8. For instance, in participant P006, the number of quality channels appeared to increase by 1 after movement tasks when using a threshold of 0.5 (Table 7). However, with a threshold of 0.8, there was a notable decrease of 17 good quality channels. Corroborating these observations, a nonparametric statistical test yielded significant results, indicating that the average number of good quality channels during E4 tasks was indeed lower than during tasks with minimal to no movement (i.e., E1, E2, E3). This underscores the impact of gross-motor movements on individual channel signal quality at higher thresholds, implying significant implications for researchers aiming to obtain reliable data during tasks involving such movements.

Collectively, this evidence hints at potential limitations in the practical application of fNIRS in naturalistic environments, suggesting a discrepancy between its perceived versatility and actual performance. Despite being positioned as a reliable method for capturing activities like biking, walking, or social interactions that are challenging for fMRI or MEG, our study suggests that fNIRS may be more susceptible to motion artifacts then commonly assumed.

## 9 Conclusion

The present study explored how hair color, hair cleanliness, light, and movement can affect the quality of an fNIRS signal. While these findings are preliminary, they provide us with justification to further explore these factors. This is especially apparent regarding hair color, and environmental light. Lighter hair colors appeared to produce better signal quality when compared to dark colors. Environmental light is also likely to play a role in affecting fNIRS signal quality, albeit to an extent that is still unclear. Thus, hair color and environmental light emerge as prime candidates deserving further investigation with a more extensive sample size to comprehensively determine the extent of their impact. In contrast, the cleanliness of one’s hair did not affect signal quality. However, given that this factor was only examined with one participant, and considering that, to the best of our knowledge, it has not been reported as a controlled variable in fNIRS-based research, further investigation into this factor could also prove beneficial. In addition, large movements such as walking, turning the head, or nodding the head were observed to deteriorate the quality of an fNIRS signal. Pertaining to this factor, future research could explore how these movements affect signal quality across different fNIRS manufacturers in order to determine which is the most resilient to these affects.

We propose that the most effective approach to achieve these future research goals would involve researchers documenting more comprehensive participant demographics, including factors related to hair, skin color, age, sex, and gender, among others. Additionally, researchers should record precise details about the lab environment during fNIRS data collection, including ambient light conditions. Furthermore, detailed accounts regarding participant exclusion based on poor signal quality, as well as the proportions of the total number of good quality channels to the number of active channels over specific brain regions, should be documented. Incorporating this information, along with signal quality analyses such as SCI measures, will enable a thorough investigation of the factors that hinder fNIRS signal acquisition.

## Supporting information

S1 Fig Good vs. bad quality channels visualized on a brain. Good quality channels (i.e., SCI ≥ 0.5) are colored green. Poor quality channels (i.e., SCI ≤ 0.5) are colored red. Yellow circles represent transmitters and blue squares represent receivers.
(PDF)

S2 Fig Locations of fNIRS channels on the brain. This image represents an SCI analysis with each of the 45 optode pairs (channels) and the brain regions they correspond to.
(PDF)
