# Supplementary figures and images for: Opening the dialogue: A preliminary exploration of hair color, hair cleanliness, light, and motion effects on fNIRS signal quality
Source: PLoS One. 2024 May 23;19(5):e0304356. doi: 10.1371/journal.pone.0304356 (PMC11115287; doi:10.1371/journal.pone.0304356)

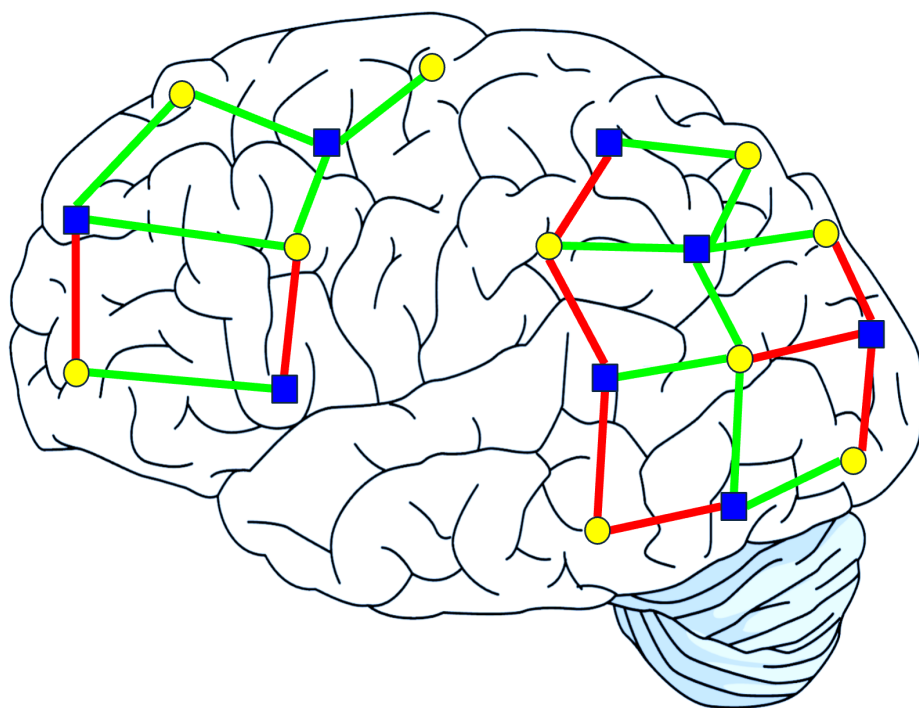

Supplement: S1 Fig — Good quality channels (i.e., SCI ≥ 0.5) are colored green. Poor quality channels (i.e., SCI ≤ 0.5) are colored red. Yellow circles represent transmitters and blue squares represent receivers. (PDF) [file pone.0304356.s001.pdf]

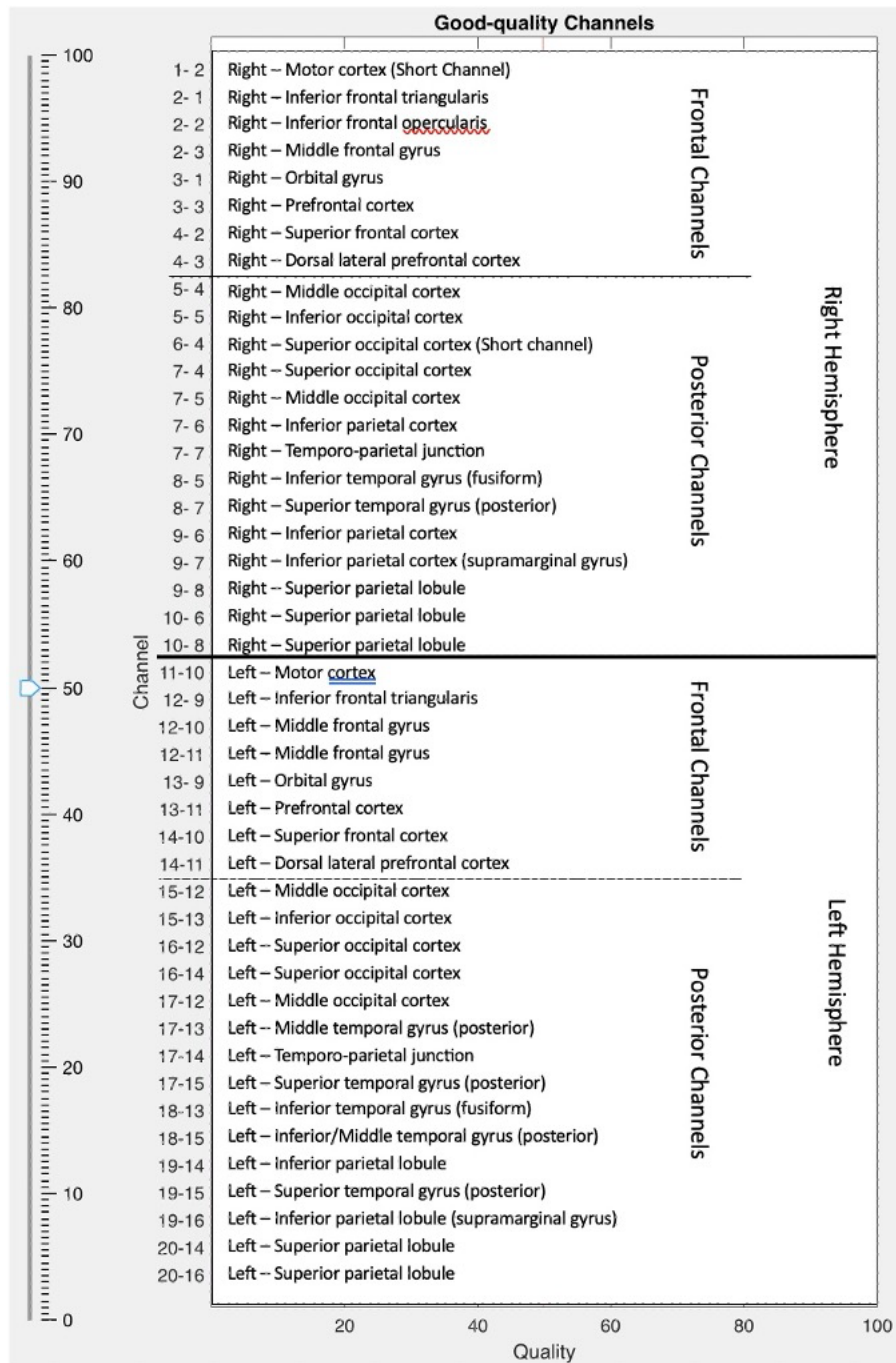

Supplement: S2 Fig — This image represents an SCI analysis with each of the 45 optode pairs (channels) and the brain regions they correspond to. (PDF) [file pone.0304356.s002.pdf]
